# Biological Activities and the Essential Oil Analysis of *Cousinia harazensis* and *C. calocephala*

**DOI:** 10.22037/ijpr.2020.114155.14697

**Published:** 2021

**Authors:** Ebrahim Salimi-Sabour, Farshad H. Shirazi, Arash Mahboubi, Faraz Mojab, Mahboubeh Irani

**Affiliations:** a *School of Pharmacy, Shahid Beheshti University of Medical Sciences, Tehran, Iran. *; b *Department of Pharmacognosy, Faculty of Pharmacy, Baqiatallah University of Medical Sciences, Tehran, Iran. *; c *Pharmaceutical Sciences Research Center (PSRC), Shahid Beheshti University of Medical Sciences, Tehran, Iran. *; d *Food Safety Research Center, Shahid Beheshti University of Medical Sciences, Tehran, Iran. *; e *Traditional Medicine and Materia Medica Research Center (TMRC), Shahid Beheshti University of Medical Sciences, Tehran, Iran.*

**Keywords:** Cousinia harazensis, Cousinia calocephala, Cytotoxicity, Anti-bacterial, Anti-fungal, Anti-malarial, Essential oil compositions

## Abstract

This research aimed to evaluate the cytotoxicity, anti-bacterial, anti-fungal and heme polymerization inhibition activities, as well as the detection of the chemical composition of essential oils and measurement of the amount of total phenol and flavonoids of *Cousinia harazensis* and *C. calocephala*. *In-vitro* growth inhibitory effects of methanol extracts on A2780, T-47D, A549 and Hep-G2 cells were evaluated by MTT assay. MIC and MBC/MFC were determined by the agar dilution method. The anti-malarial activity of herbs was assessed with an inhibition test of heme detoxification (ITHD). Total phenol and flavonoids content measured by Folin-Ciocalteu method. The essential oils from two herbs were extracted by hydro-distillation, and GC/MS analyzed their compositions.

Cell studies against selected cell lines growth in MTT assay were related to *C. harazensis* on Hep-G2 with IC_50_ of 4.521 µg/mL. The MIC of anti-bacterial and anti-fungal effects is related to *C. harazensis* extract on *Staphylococcus epidermidis* and *Aspergillus fumigatus* with 15.62 and 62.5 mg/mL, respectively. Both extracts do not have anti-malarial activity. *C. harzensis *content was richer in total phenol and flavonoids rather than the other herb. *m*-benzyl benzyl alcohol (46.7%) and butyl phthalate (14.7%) are the major compounds of *C. harazensis*; main components of *C. calocephala* are 3-methyl-tetrahydrofuran (24.6%) and oleic acid (15.4%).

In conclusion, *C. harazensis* with more phenol and flavonoids content showed better results in terms of biological activities.

## Introduction

Herbs are a valuable source of various novel pharmacognostic compounds. Despite some studies, the biological effects of many plants’ extracts and essential oils have not yet been examined, and new species should be screened to discover new pharmacological effects (1).

Flora of Iran is one of the richest floras globally and has 1800 endemic species from 8000 taxa. The genus *Cousinia* with 600-700 species, is the largest genera of flowering plants in Central and Western Asia (2). After *Astragalus*, *Cousinia* with approximately 240 species, is considered as the second largest genus in flora of Iran (3). *Cousinia harazensis* Rech.f. (CH) and *C. calocephala *Jaub. & Spach (CC) are endemic to Iran (4). There are few studies about biological activities and phytochemical research of the genus *Cousinia,* thus, in this article, some biological effects of the herbs were studied.

Regarding the side effects, high toxicity, and drug resistance observed with current anti-cancer and anti-microbial agents, especially synthetic drugs, many kinds of research have been oriented toward natural components, especially from plants; so in this work, we studied cytotoxicity, anti-bacterial, anti-fungal and anti-malarial activities and essential oil analysis of CC and CH for the first time. 

Despite the advances in biomedical research and innovative technologies, cancer is a growing public health problem; the estimated number of new cancer cases in the United States in 2020 is over 1.8 M (5). One study in 2019 showed that chemical components of *C. davisiana*, *C. foliosa*, *C. ramosissima* and *C. stenocephala* were examined against Colo205 (human colon carcinoma) and A549 (human non-small cell lung cancer) cell lines. The best result was related to *C. stenocephala *(IC_50 _for Colo205: 130 and for A549: 990 (μg/mL)) and ψtaraxasterol (IC_50 _for Colo205: ≥100 and for A549: ≥100 (μg/mL)) (6). Sajjadi *et al.* studied the cytotoxic activity of *C. verbascifolia *against OVCAR-3 and HT-29 cancer cells. The IC_50 _values against OVCAR-3 and HT-29 cells were 119 - 190 and 118 - 194 μg/mL, respectively (7). Sajjadi *et al.,* in another study, isolated two phenolic components from the *C. verbascifolia *that had moderated cytotoxicity against OVCAR-3 and HT-29 cell lines (8).

Iranshahy *et al.* reported some highly oxygenated sesquiterpene lactones from *C. aitchisonii* with acceptable IC_50 _against MCF-7, MCF-7/MX, PC3, HL-60, Jurkat and HEK (all below 15 μg/mL) (9).

Infections**-**induced death is one of the ten first mortality factors in the world (10). Many new anti-microbial and anti-parasite agents have been discovered and marketed. Still, because of the genetic ability to change gene expression and other mechanisms, resistance to these drugs is growing (11, 12).

The importance of resistance to anti-microbial and anti-parasite drugs has not only emerged in hospitals but also occurred in community environments (11, 12) and it can be so harmful, especially for patients with the suppressed immune system. According to new infections**,** it can lead to an increase in hospitalization (13).

Methanol extracts of *C. lachnosphaera* affect *Pseudomonas aeruginosa* and* Staphylococcus aureus; *and *C. turkmenorum* against *S. aureus* (14). Anti-bacterial activity of *C. onopordioides*, *C. rechingerae*,* C. hypopolia*,* C. sulabadensis*,* C. phyllocephala*,* C. smirnovii *and *C. verbascifolia* were assessed against five selected bacteria (15). Amiri *et al.* studied anti-bacterial activity from the root of *C. microcarpa* against some selected Gram-positive and Gram-negative bacteria. The best result of the disk diffusion method was for methanol extract with a 19 mm zone against *P. aeruginosa* (16). 

In anti-fungal activity, Bahraminejad *et al.* evaluated a methanol extract of *C. stenocephala* and found that this extract cannot inhibit the growth of *Fusarium oxysporum* (17). In another study, none of the selected species of *Cousinia* were reported to have activity against *Candida albicans* (14). Bahraminejad *et al.* assessed the anti-fungal potency of *C. stenocephala* against *Phytophtora drechsleri*, *Pythium aphanidermatum* and *Rhizoctonia solani* and the results showed that the methanol extract has acceptable effects (18).

However, no studies have addressed the anti-malarial activity of *Cousinia****,*** and the present study is the first assessment of inhibition test of heme detoxification from the species.

Essential oils are in some higher plants, and they have some applications such as carminative, flavor agents, anti-microbial activities, *etc*. (19, 20). This research aims to render information to report the oil constituents of CH and CC. 

## Experimental

*Plant Materials*

Aerial parts of CH and CC were collected from Lasem Area (Mazandaran Province, Iran) in July 2018. Voucher specimens were deposited in the Central Herbarium of Tehran University, Tehran, Iran, by No: 48400 and 48401, respectively.

A. Rastegar A. identified the herbs at Kurdistan Agriculture and Natural Resources Research and Education Center, Sanandaj, Iran. The herbs were cleaned and dried, and then they were grounded to a fine powder.

*Extraction Procedure*

One-hundred grams of powdered parts of each herb were macerated in 300 mL methanol 80% (Chem-Lab, Belgium), and they were shaken for 24 h (GFL, Germany) at room temperature and repeated (X 3). Then**,** solvents were evaporated using a rotary evaporator (Heidolph, Germany). The concentrated extracts were dried in dry-oven at 40 °C to remove the remained solvents. The resulting extracts were kept in sterile containers at 4 °C for further tests.

*Cell Culture*

A2780 ECACC 93112519 (Human ovarian carcinoma), T-47D ATCC HTB-133 (Ductal carcinoma), A549 ATCC CCL-185 (Human lung carcinoma) and Hep-G2 ATCC HB-8065 (Hepatocellular carcinoma) were purchased from Pasteur Institute (Tehran, Iran). All cell lines were grown in RPMI 1640 medium (Gibco, USA) supplemented with 10% heat-inactivated FBS (Gibco, USA), 100 μg/mL penicillin (Panbiotech, Germany) and 100 μg/mL streptomycin (Panbiotech, Germany). The cultured cells were retained at 37 C in a 5% CO2-incubator (Heraeus, Germany). All cellular experiments were performed in triplicate. Cell viability was tested by cell count using trypan blue (Gibco, USA) stain 0.4% under a light-inverted microscope (Leica, Germany). 3500 cells were seeded in 96 well cell culture plates (SPL, South Korea). The cells were treated for 48 h by different concentrations of: 2.3437, 4.6875, 9.375, 18.75, 37.5, 75, 150, 300 and 600 μg/mL of each extract. The cytotoxicity effects of each extract was evaluated by 3-(4, 5-dimethylthiazol-2-yl)-2, 5-diphenyltetrazolium bromide (MTT) (Merck, Germany) assay (21).

*MTT assay*

As mentioned before, after 48 h, 20 μL of MTT (5 mg/mL) was added to each well and incubated for 4 h. The medium containing MTT was then aspirated, and 100 μL of dimethyl sulfoxide (DMSO) was added to each well. Then**,** the plates were gently agitated until the formed formazans were dissolved. The absorbance of each well was read using a plate reader (Rainbow, Australia) at 570 nm, subtracting the absorbance at 650 nm as a reference (there is no absorbance by MTT at 570 nm). Sample groups were compared by ANOVA with Bonfferoni correction, and non-linear regression was applied to estimate the IC_50_ of each extract.

*Bacterial Strains and Culture Media*

Five Gram-positive bacteria including *Staphylococcus aureus* ATCC 6538, *S.* *epidermidis* ATCC 12228, *Kocuria rhizophila* ATCC 9341, *Bacillus* *subtilis* ATCC 6633 and *B.* *cereus* ATCC 1247 and five Gram-negative bacteria, *Escherichia coli* ATCC 8739, *Pseudomonas aeruginosa *ATCC 9027, *Salmonella* *enterica* ATCC 19430, *Klebsiella pneumoniae* ATCC 10031 and *S.* *typhimurium* ATCC 14028 were purchased from Iranian Research Organization for Science (IROST) and Technology, Tehran, Iran and were cultured in Mueller-Hinton Agar (MHA) medium and incubated at 37 °C for 18-24 h.

*Determination of Inhibition Zone*

Pre-evaluation of the anti-bacterial activity of the extracts was studied by the cup-plate method. In short, bacterial suspension with turbidity equal to 0.5 McFarland standards (1 × 10^8^ CFU/mL) was prepared. The MHA plates were streaked by this suspension. The wells with diameters 8 mm were filled with 100 μL of different concentrations of the extracts, including 500, 250, 125, 62.5 mg/mL. The solvent was also added in a well as a negative control. The plates were incubated at 37°C for 18-24 h. The cup-plate method was performed 3 times, and the average diameters of inhibition zones for each concentration were determined.

*Determination of Minimum Inhibitory Concentration (MIC)*

MIC was determined by the micro-dilution method according to the Clinical and Laboratory Standards Institute (CLSI) recommended method (22). CH was mixed with Muller-Hinton Broth (MHB) and Tween 80 (80: 20)**,** the solvent was used as a negative control**,** and CC was mixed with MHB to make homogeneous concentrations: 500, 250. 125, 62.5, 31.25, 15.62, 7.81, 3.90, 1.95, 0.97 and 0.48 mg/mL (One row was considered for evaluation of the extract sterility). The bacterial suspensions corresponding to 0.5 McFarland were made and diluted 20 times with sterile normal saline. Ten microliters of each bacterial suspension was added to each well of the micro-plate. One row without plant extract was considered as growth control. The micro-plates were incubated at 37 °C for 24 h. The lowest concentration with no visible growth was considered as minimum inhibitory concentrations (MICs) of the extracts. MIC evaluation was repeated 2 times more. 

*Determination of Minimum Bactericidal Concentration (MBC)*

Twenty microliters of each well**,** in which no growth was added to MHA medium and plates were incubated at 37 °C for 18-24 h. 

*Fungal Strains and Culture Media*

*Candida albicans* ATCC 10231 and *Aspergillus fumigatus* PTCC 5009 were purchased from IROST, Tehran, Iran. 

*C. albicans *was cultured on Sabouraud Dextrose Agar (SDA) medium and incubated at 37 °C for 24-48 h. *A. fumigatus* was activated at 27 °C for 24-48 h in a similar medium and condition. 

*Preparation of Fungal Suspensions*

*Candida albicans* (yeast)

Standard suspension yeast was prepared by broth micro-dilution method as described by Clinical and Laboratory Standards Institute (CLSI) guidelines, document M27-S3 (23). The RPMI 1640 medium (Gibco, USA) was used. The optical turbidity of *C. albicans* suspension was adjusted to 75 to 77% at a wavelength of 530 nm, and 1000 times more dilutions were performed with the mentioned medium. The final inoculum concentration was adjusted to 0.5 × 10^3^ to 2.5 × 10^3^ CFU/mL (24). 

*Aspergillus fumigatus *(Mold)

To prepare a standard suspension, CLSI guideline, document M38-A2 was used, and the optical turbidity of this mold suspension was adjusted to 78 to 82% at a wavelength of 530 nm, 50 more dilutions were done with RPMI 1640, and final inoculum concentration was adjusted to make about 5 × 10^4^ CFU/mL (25).

*Determination of Inhibition Zone*

Pre-evaluation of anti-fungal activities of these methanol extracts was performed via the disk diffusion test and based on the CLSI method (26). 100 μL of each standard suspension was added on the surface of the plates filled with SDA medium and then speared in three roundabouts with a sterile loop. Then**,** the disks with diameters equal to 6.4 mm were used. These disks were smeared with different concentrations of the extracts, including 500, 250, 125, 62.5 mg/mL dissolved in distilled water and tween 80 (D/T) (8:2), D/T was used as a negative control. All plates were incubated for 24-48 h. The disk diffusion method was performed 3 times, and the average diameters of inhibition zones for each concentration were determined.

*Determination of Minimum Inhibitory Concentration (MIC)*

To evaluate the MIC value based on CLSI methods, CH was mixed with RPMI 1640 and Tween 80 (80:20) and this solvent was used as a negative control and CC was mixed completely with RPMI 1640 to make homogeneous concentrations: 500, 250. 125, 62.5, 31.25, 15.62, 7.81, 3.90, 1.95, 0.97 and 0.48 mg/mL (One row was considered for evaluation of each extract sterility). One-hundred microliters of each standard suspension was added to each well of micro-plate. One column without plant extract was considered as growth control. The plates with *C. albicans* suspension were incubated at 27 °C, and plates that contained *A. fumigatus* were incubated at 37 °C for 24-48 h (X 2). 

*Determination of Minimum Fungicidal Concentration (MFC)*

Ten μL of each well content in which no growth observed was added to the SDA medium, and plates were incubated at mentioned conditions.

*Inhibition Test of Heme Detoxification*

Inhibition of β-hematin formation was used for the inhibition test of heme detoxification as a spectrophotometric assay. Hemin Chloride (Merck, Germany) was dissolved in DMSO (Merck, Germany). The solution was diluted (1:1) freshly with 1 M acetate buffer (pH 4.8). Diluted hemin (60 μg/mL^-1^), tween 20 (0.012 g/L), and samples (200 μg.mL^-1^) were mixed in each well of a 96-well plate with a ratio of 9:9:2, respectively. The herbs and chloroquine diphosphate were used as negative and positive controls, respectively. The plates were incubated at 60 °C for 24 h (X 3). Finally, the absorbance was recorded with an ELISA reader at 405 nm. The results were calculated as the percentage of heme detoxification inhibition (27).

*Total phenolic content assay*

Total amounts of phenolic compounds in each extract were determined by Folin-Ciocalteu colorimetric method. A calibration curve obtained with Gallic acid (Merck, Germany) as a standard was drawn, and phenolic compounds in the extracts were carried out in triplicate and calculated according to this line diagram equation. Total phenolic contents were expressed as a percentage of Gallic acid equivalents in dry extract matter (28).

*Total flavonoid content assay*

The total amount of flavonoid compounds was assessed by the AlCl_3_ colorimetric method (28). A calibration curve obtained with rutin (Merck, Germany) as a standard was drawn, and flavonoid compounds in the extracts were carried out in triplicate and calculated according to this line diagram equation. Total flavonoid contents were expressed as a percentage of rutin equivalents in dry extract matter.

*Isolation of the Essential Oil*

The essential oils were obtained by the hydro-distillation method by using a Clevenger-type apparatus for 3 h. The essential oils were dried over anhydrous sodium sulfate and kept in a sealed dark vial at 4 °C until analysis.

*GC-MS Analysis*

GC-MS analysis was carried out on a Thermofinnigan GC-MS instrument equipped with a DB-5 fused silica column (30 m X 0.5 µm, film thickness 0.33 mm). The oven temperature program was raised from 50 °C to 100 °C at a rate of 5 °C/min, and then raised 100 °C to 150 °C for 2 min at a rate of 5 °C/min; then set up 150 °C to 200 °C for 2 min at a rate of 5 °C/min; then raised 200 °C to 230 °C for 2 min at a rate of 5 °C/min and then raised 230 °C to 260 °C for 2 min at a rate of 10 °C/min and held for 2 min. Helium was used as the carrier gas at a flow rate of 1.5 ml/min; The splitless mode was performed. The quadrupole mass spectrometer was scanned over the 35-375 AMU with an ionizing voltage of 70 eV and an ionization current of 150 mA.

The essential oil components were identified by comparing their mass spectra with those reported in the literature and presented in the internal reference mass spectra library (Wiley/NIST).

## Results and Discussion

*Yield of Extraction*

The extraction yields of *Cousinia harazensis* and *C. calocephala* were 10.6 and 10.4%, respectively.

*Analysis of Essential Oils*

The essential oils were colorless and characteristic odor. Analysis of oil from CH and CC identified 9 and 15 compounds, representing 98.9 and 63.9% of the total constituents, respectively. The identified compounds and their percentage are summarized in Tables 1 and 2.

*m*-benzyl benzyl alcohol (46.7%), butyl phthalate (14.7%), oleic acid (14.2%) and palmitic acid (11.2%) are some of the major compounds of CH; main components of CC are 3-methyl-tetrahydrofuran (24.6 %) and oleic acid (15.4%). 

The main class of the compounds in herbs is presented in Table 3. According to this table, CC has monoterpenes and sesquiterpenes (10.4 and 5.9%).

*Evaluation of Cytotoxicity*

Different results from two extracts against A2780, T-47D, A549 and Hep-G2, were obtained. According to the results of CH, apart from the effect of methanol extract against A2780 that was relative and transient proliferative (RTP effect), other results showed that the best cytotoxic effect was related to the effect of CH against Hep-G2 (IC_50_: 4.52 ± 3.19 µg/mL, p < 0.05).

Methanol extract of CC has no effects on T-47D and A549, but CC has a proliferative effect against A2780, and the EC_50_ of it was 8.03 ± 4.49 µg/mL. All MTT assays are presented in Table 4.

According to Balantyne *et al*. about the classification of the cytotoxicity for natural ingredients, based on this article, the result of CH against Hep-G2 is considered as a potentially very toxic extract. The effect of CH on T-47D and A549 might be classified as potentially toxic (29).

*Anti-Bacterial Results*

*Zone of Inhibition Diameters*

Pre-evaluations of anti-bacterial activity of the samples using the cup-plate technique are presented in Tables 5 and 6. These results showed that the diameters of inhibition zones of the samples dilutions were increased concerning the concentration of the extracts except in CH. Results showed that in the concentration of 500 mg/ml against *P. aeruginosa,* the diameter of the inhibition zone was decreased. The best result of this item was related to the effect of CH (Conc. 500 mg/mL) against *S. epidermidis* with 31.3 ± 0.70 mm inhibition zone diameter.

*Result of MIC Value*

In order to determine the MIC values, two-fold serial dilutions from concentrations of 500 to 0.48 mg/mL were used. Between both Gram-positive and Gram-negative strains, CH inhibits in lower concentration than CC. The lowest concentration that could inhibit the growth of bacteria was related to CH extract on *S. epidermidis* with a 15.62 mg/mL MIC value (Table 7).

*Result of MBC Value*

According to Table 8, both extracts can kill Gram-positive bacteria in the same concentrations; however, by comparing the effect of these two extracts on Gram-negative bacteria, it has been found that CH has the best results, especially its effect on *P. aeruginosa* and *S. enterica* with MBC value 62.5 mg/mL.

*Anti-Fungal Results*

*Zone of Inhibition Diameters*

Pre-evaluation of anti-fungal activity of the extracts was performed by using disk diffusion technique. These results showed that the extracts in each concentration could not inhibit the growth of fungal strains. 

*Result of MIC Value*

In order to determine the MIC values, two serial dilutions from the concentration of 500 to 0.48 mg/mL were used (Table 9). CH had the most anti-fungal effect against *A. fumigatus* with MIC 62.5 mg/mL.

*Result of MFC Value*

Similar MIC results are obtained for MFC, and CH proved better than CC. The results are shown in Table 10. 

*Result of Inhibition Test of Heme Detoxification (ITHD)*

In this method, if the percentage of ITHD was more than 90%, the assay would be considered as positive, and values less than 90% indicated a negative result. The results of the two extracts do not have anti-malarial activity in this mechanism.

*The Results of Total Phenol and Flavonoid content assays*

The total phenol compounds was evaluated by Folin-Ciocalteu colorimetric method, which showed that CH and CC were 7.45 and 4.57%, total flavonoid content assays were present 4.57 and 2.73%, respectively.

**Table 1:** Chemical constituents of the essential oils of CH

**No.**	**Compound**	**RT**	**(%)**
**1**	1-methoxy-4-(1-methoxy phenyl)benzene	8.78	2.7
**2**	1,4 diphenylbut-3-ene-2-ol	13.2	3.6
**3**	Lauric acid 2,3-diacetoxy propyl ester	35.86	0.4
**4**	Butyl phthalate	37.33	14.7
**5**	Palmitic acid	38.29	11.6
**6**	Oleic acid	42.31	14.2
**7**	Stearic acid	42.87	4.3
**8**	Ethyl linoleate	43.76	0.7
**9**	*m*-benzyl benzyl alcohol	45.07	46.7

RT: Retention times.

**Table 2:** Chemical constituents of the essential oils of CC

**No.**	**Compound**	**RT**	**(%)**
**1**	3-methyl-tetrahydrofuran	4.51	24.6
**2**	β-ocimene	11.99	1.3
**3**	α-pinene	13.45	5.3
**4**	Decanal	16.93	2.5
**5**	Thymol	19.37	2.9
**6**	2,4-decadienal	20.27	2.0
**7**	Limonen dioxide	22.25	0.9
**8**	Curcumene	25.45	1.1
**9**	1-tetradecanol	26.16	2.0
**10**	α-Gurjunene	30.34	4.8
**11**	Hexadecanal	32	1.5
**12**	Neophytadiene	35.35	3.5
**13**	Oleic acid	38.13	15.4
**14**	Pentacosane	45.92	4.5
**15**	Heptacosane	49.4	2.0

RT: Retention times.

**Table 3:** Category of major components in CH and CC (%).

**Major Components**	**CH**	**CC**
Monoterpenes	-	10.4
Sesquiterpenes	-	5.9
Diterpenes	-	3.5
Aliphatic components	-	14.5
Fatty acids	30.1	15.4
Phenolic compounds	53.0	-
Ester compounds	15.8	-
Other compounds	-	14.2

**Table 4:** The results of cytotoxicity by MTT assay method (µg/mL), numbers represent mean ± SD

**No.**	**Cell line type**	**CH**	**CC **
**1**	A2780	RTP effect	EC_50_: 8.03 ± 4.49
**2**	T-47D	IC_50_: 32.21 ± 8.58	No effect
**3**	A549	IC_50_: 31.68 ± 10.9	No effect
**4**	Hep-G2	IC_50_: 4.52 ± 3.19	RTP effect

**Table 5:** The mean (±SD) of inhibition zone diameter of extracts in Gram-positive bacteria (mm), n = 3

Extract	Concentration of extract (mg/mL)	*S. aureus*	*S. epidermidis*	*M. luteus*	*B. subtilis*	*B. cereus*
CH	500 250 125 62.5	24.5 ± 0.70 23 ± 1.41 20.5 ± 2.12 17 ± 1.41	31.5 ± 0.70 30.5 ± 0.70 29.5 ± 0.70 21.5 ± 2.12	28.66 ± 1.52 25.33 ± 0.57 21.66 ± 1.52 19.33 ± 0.57	17.5 ± 0.70 16.5 ± 0.70 15.5 ± 0.70 14.5 ± 2.12	25.5 ± 0.70 24.5 ± 0.70 22 ± 0 19 ± 2.82
CC	500 250 125 62.5	0 0 0 0	17.66 ± 0.57 14.66 ± 0.57 11.66 ± 0.57 11 ± 1	14 ± 0 13 ± 1.73 0 0	11 ± 0 0 0 0	12 ± 1.41 0 0 0

**Table 6:** The mean (±SD) of inhibition zone diameter of extracts in Gram-negative bacteria (mm), n = 3

Extract	Concentration of extract (mg/mL)	*E. coli*	*P. aeruginosa*	*S. enterica*	*K. pneumonia*	*S. typhimurium*
CH	500 250 125 62.5	22.00 ± 1 21.66 ± 0.57 19.33 ± 1.15 16.33 ± 0.57	20.66 ± 1.15 22.66 ± 2.08 19.66 ± 2.08 18.66 ± 2.08	15.66 ± 0.57 14.33 ± 0.57 0 0	12.66 ± 0.57 0 0 0	18.33 ± 1.15 16.00 ± 0 14.33 ± 1.15 10.66 ± 1.15
CC	500 250 125 62.5	0 0 0 0	19.33 ± 0.57 18.33 ± 0.57 18.00 ± 1 16.00 ± 1	16.33 ± 1.15 13.66 ± 1.15 0 0	23.00 ± 1.73 18.33 ± 1.52 16.33 ± 1.52 14.66 ± 0.57	21.00 ± 0 18.33 ± 0.57 16.66 ± 1.15 15.33 ± 0.57

**Table 7:** The mean MIC of extracts in bacteria (mg/mL), n = 2

**Type of bacteria**	**Bacteria**	**CH**	**CC **
Gram positive	*S. aureus*	31.25	62.5
	*S. epidermidis*	15.62	31.25
	*K. rhizophila*	62.5	62.5
	*B.* *subtilis*	62.5	62.5
	*B.* *cereus*	125	125
Gram-negative	*E. coli*	62.5	125
	*P. aeruginosa*	31.25	62.5
	*S.* *enterica*	62.5	62.5
	*K. pneumoniae*	62.5	125
	*S typhimurium*	62.5	125

**Table 8:** The mean of minimum bactericidal concentration (MBC) of extracts (mg/mL), n = 3

**Type of bacteria**	**Bacteria**	**CH**	**CC **
Gram-positive	*S. aureus*	125	125
	*S. epidermidis*	62.5	62.5
	*K. rhizophila*	62.5	62.5
	*B.* *subtilis*	500	500
	*B.* *cereus*	125	125
Gram-negative	*E. coli*	250	250
	*P. aeruginosa*	6.5	125
	*S.* *enterica*	62.5	125
	*K. pneumonia*	250	250
	*S typhimurium*	250	500

**Table 9:** The mean MIC of extracts in different fungal (mg/mL), n = 2

**Type of fungal**	**Fungal**	**CH**	**CC **
Yeast	*C. albicans*	250	500
Mold	*A. fumigatus*	62.5	500

**Table 10:** The mean of minimum fungicidal concentration (MFC) of extracts in different fungal (mg/mL), n = 3

**Type of fungal**	**Fungal**	**CH**	**CC **
Yeast	*C. albicans*	250	500
Mold	*A. fumigatus*	62.5	Not Detected

## Discussion

In this study, we assessed cytotoxicity, anti-bacterial, anti-fungal and inhibition test of heme detoxification method for anti-malarial activities of CH and CC. Assessment of anti-malarial activity of *Cousinia* species was studied for the first time. 

According to the results of the MTT assay, different cytotoxicity and proliferative effects were seen from these extracts. Except for A2780, the results of the cytotoxicity effect of CH were significant against T-47D, A549 and Hep-G2. Despite the result of CH on these cell lines, CC did not show any effect on T-47D and A549. In contrary, CH presented a proliferative effect on A2780 and RTP effect on the Hep-G2 cell lines. 

The result of our study on CH on A549 is comparable with *C. davisiana*, *C. foliosa*, *C. ramosissima* and *C. stenocephala *(6) that has shown the highest cytotoxicity for CH than others. No other investigations were found to compare our results for the herbal extracts against these cell lines. 

About the result of the anti-bacterial and anti-fungal activities of two methanol extracts, the results show that CH is more effective than another extract. Both CH and CC had better effects on selected bacteria strains rather than *C. lachnosphaera* and *C. turkmenorum* (14). These results show that CH can inhibit all of the Gram-positive and Gram-negative bacteria. However, *C. lachnosphaera* affects *P. aeruginosa* and *S. aureus*, while *C. turkmenorum *was only effective on *S. aureus*. None of the extracts in these two studies can inhibit the growth of *C. albicans*.

Amiri *et al.* studied the anti-bacterial activity of *C. microcarpa* (16). Looking at the inhibition zone diameter results of his study, CH with 31.5 mm has the best effects against *P. aeruginosa* compared to *C. microcarpa* with 19 mm. 

To our knowledge, few studies been have been conducted on the chemical compositions of *Cousinia* species so far. According to Tables 1 and 2, two compounds, including 1-methoxy-4-(1-methoxy phenyl) benzene and m-benzyl benzyl alcohol, are similar in structure. The other compound, ethyl linoleate was found in the essential oil of *Daphne sericea* (3.9%), *Gardenia jasminoides* (1.3%) and *Datura metel* (17.4%), too (30-32). Some other compounds, including oleic acid, palmitic acid and stearic acid were detected in *Centaurea solstitialis*, *Moringa oleifera* and *Citrus *spp*.* peel (33-35). α-pinene, as we reported 5.3% in CH, is one of the famous compounds, and it is found in turpentine very much (36). This compound has different biological effects such as anti-peptic ulcer, anti-malarial and anti-bacterial activity (37-39). According to Tekin *et al.*, fatty acids/fatty acid esters (46.1%) and esters (20.2%) are the integral ingredients of *C. savasica,* and the results indicated the similarity between the class of compounds of *C. savasica* and *C. harazensis* with varying components (40).

In conclusion, the biological research indicates that *C. harazensis* has considerable potential and justifiable of folk and traditional use. The biological effects can make contributions to the isolation of active ingredients. These effects can be attributed to their phenol and flavonoids contents.
